# Assessment of the Reporting Quality of Randomized Controlled Trials on the Treatment of Diabetes Mellitus with Traditional Chinese Medicine: A Systematic Review

**DOI:** 10.1371/journal.pone.0070586

**Published:** 2013-07-24

**Authors:** Ping Wang, Qin Xu, Qi Sun, Fang-fang Fan, Xue-rui Guo, Fei Guo

**Affiliations:** The Affiliated Traditional Medical Hospital, Xinjiang Medical University, Urumqi, Xinjiang Uygur Autonomous Region, China; Georgia Health Sciences University, United States of America

## Abstract

**Background:**

After the publication of the CONSORT 2010 statement, few studies have been conducted to assess the reporting quality of randomized clinical trials (RCTs) on treatment of diabetes mellitus with Traditional Chinese Medicine (TCM) published in Chinese journals.

**Objective:**

To investigate the current situation of the reporting quality of RCTs in leading medical journals in China with the CONSORT 2010 statement as criteria.

**Methods:**

The China National Knowledge Infrastructure (CNKI) electronic database was searched for RCTs on the treatment of diabetes mellitus with TCM published in the Journal of Traditional Chinese Medicine, Chinese Journal of Integrated Traditional & Western Medicine, and the China Journal of Chinese Materia Medica from January to December 2011. We excluded trials reported as “animal studies”, “in vitro studies”, “case studies”, or “systematic reviews”. The CONSORT checklist was applied by two independent raters to evaluate the reporting quality of all eligible trials after discussing and comprehending the items thoroughly. Each item in the checklist was graded as either “yes” or “no” depending on whether it had been reported by the authors.

**Results:**

We identified 27 RCTs. According to the 37 items in the CONSORT checklist, the average reporting percentage was 45.0%, in which the average reporting percentage for the “title and abstract”, the “introduction”, the “methods”, the “results”, the “discussion” and the “other information” was 33.3%, 88.9%, 36.4%, 54.4%, 71.6% and 14.8%, respectively. In the Journal of Traditional Chinese Medicine, Chinese Journal of Integrated Traditional & Western Medicine, and the China Journal of Chinese Materia Medica the average reporting percentage was 42.2%, 56.8%, and 46.0%, respectively.

**Conclusions:**

The reporting quality of RCTs in these three journals was insufficient to allow readers to assess the validity of the trials. We recommend that editors require authors to use the CONSORT statement when reporting their trial results as a condition of publication.

## Introduction

Randomized controlled trials (RCTs), when appropriately designed, conducted, and reported, represent the gold standard for evaluating healthcare interventions. However, randomized trials can yield biased results if they lack methodological rigor. To accurately assess a trial, readers of a published report require complete, clear, and transparent information on its methodology and findings. Unfortunately, assessments frequently fail because authors neglect to provide clear and complete descriptions of such critical information [1].

Reporting quality assessment is therefore the first stage in a critical literature review. In 1996, the Consolidated Standards for Reporting of Trials (CONSORT) group produced the CONSORT statement, an evidence-based approach to help improve the quality of reporting RCTs. The group published a revised statement in 2001. The methodological factors included in the CONSORT statement were selected due to empirical evidence indicating their importance. The CONSORT statement has been supported by a growing number of medical and healthcare journals and editorial groups, including the International Committee of Medical Journal Editors (ICMJE), the Council of Science Editors (CSE), and the World Association of Medical Editors (WAME) [2]. Subsequently, the expanding body of methodological research reported the refinement of CONSORT 2010. Over the past 16 years, a number of CONSORT recommendations (including updates and extensions) for the publication of RCTs have been developed.

Since their introduction, the quality of published RCTs has improved significantly in journals endorsing the CONSORT criteria. For example, analyses of the cardiothoracic and general surgery literature indicate substantial improvements in the reporting of RCTs after the CONSORT criteria were endorsed by their principal journals [3].

Traditional Chinese medicine (TCM) plays an important role in maintaining the health of the Chinese population (particularly before the introduction of western medicine into China) and its benefits are gradually being recognized worldwide [4]. The rapid increase in diabetes mellitus is becoming a serious health threat in all parts of the world. With its distinctive traditional medical opinions and natural medicines mainly originating from herbs, TCM has shown promise in clinical practice for the treatment of diabetes mellitus and its complications. Based on a large number of chemical and pharmacological research studies, numerous bioactive compounds have been found in Chinese medicinal plants for the treatment of diabetes [5]. Traditional Chinese herbal medicines have been used for a long time to treat diabetes, and many controlled trials have been carried out to investigate their efficacy [6].

Although the quality of reporting in RCTs in the medical sciences has been discussed, the quality of reporting in RCTs on the treatment of diabetes mellitus with TCM published in the Chinese language has not yet been assessed following publication of the CONSORT statement (2010 version). The aim of this study was to assess the completeness of reporting RCTs evaluating TCM in the treatment of diabetes mellitus published in three core Chinese medical journals based on the CONSORT 2010 checklist [7], and to provide recommendations for improving them in the future.

## Materials and Methods

### Search Strategy

The China National Knowledge Infrastructure (CNKI) electronic database was used to search all articles published between 01 January and 31 December 2011 reporting an RCT (i.e., a trial in which the assignment of participants to interventions was described by the words random, randomly, randomized, or randomization) in the full text. We only obtained the full text of RCTs on the treatment of diabetes mellitus with TCM, which were published in 2011, took place in China and included Chinese citizens.

The following search equation was used: “random, randomly, randomized, or randomization” AND “diabetes mellitus”(in the full text) AND (“2011/01/01″[PDat]: “2011/12/31″[PDat])) OR “Journal of Traditional Chinese Medicine” OR “Chinese Journal of Integrated Traditional & Western Medicine” OR “China Journal of Chinese Materia Medica”.

### Selection of Journals and RCTs

We selected three CSCD (Chinese Science Citation Database) (2011–2012) -indexed Chinese medical journals (Journal of Traditional Chinese Medicine, Chinese Journal of Integrated Traditional & Western Medicine, and the China Journal of Chinese Materia Medica) with a top ranking impact factor as our source to identify RCTs. We selected these three core journals as they are thought to publish the majority of RCTs with TCM and each trial is reported in the Chinese language.

All potentially eligible full text articles were collected. These articles were then evaluated using the eligibility criteria.

We chose this time period to better assess the reporting quality of RCTs on the treatment of diabetes mellitus with TCM, which was after the publication of the CONSORT 2010 statement.

### Eligibility Criteria

Two reviewers searched the three journals and selected potentially relevant articles after screening the titles and abstracts independently. In the case of uncertain eligibility, the full text was screened.

The clinical studies included in this review met the following criteria: 1) the report was published between 01 January and 31 December 2011; 2) the report was published as an original article of a RCT; 3) adult participants (18 years or older) with diabetes mellitus; and 4) TCM intervention including Chinese herbal medicines and Chinese proprietary medicines.

We excluded the trials reported as “animal studies”, “in vitro studies”, “case studies”, or “systematic reviews”.

For all remaining articles, the full text of the article was obtained and reviewed.

### Data Extraction

The quality of reporting in RCTs was evaluated using a 37-item modified CONSORT checklist. The revised 25-item CONSORT checklist contains some items with multiple parts. These were separated into individual items in our modified checklist.

For the purpose of this study, the reviewers underwent systematic training. Initially, they received the “Revised CONSORT Statement for Reporting Randomized Trials: Explanation and Elaboration” [8] document which provides the meaning and rationale for each checklist item and examples of good reporting practice. All authors received training in research methodology for three consecutive months, including the studied CONSORT Statement 2010.

Prior to data extraction, as a calibration exercise, both authors independently evaluated two reports on RCTs that were not included into this study. Where differences in the interpretation of an item occurred, discussion ensued and consensus among the investigators was reached allowing clarification on how to score the item.

After training, data from the included trial reports were retrieved, independently and in duplicate, by the two authors and added to tables containing the 37 items to be assessed (Table 1, Table 2, Table 3, Table 4, Table 5 and Table 6). Discrepancies were resolved by consensus between those in charge of the primary evaluation and then checked under the supervision of QS. Once we had ensured consistency in the interpretation of the data extraction form we carried out double data extraction on all the remaining trials. No attempts were made to contact trial authors as the clarity and completeness of reporting were being assessed specifically.

**Table 1:** The average reporting percentage for the title and abstract section of the CONSORT checklist in the 3 core journals.

Secti-on/To-pic	Item No	Checklist item	JTCM Yes(%)N = 21	CJITWM Yes(%) N = 5	CJCMMYes(%) N = 1	Total Yes(%) N = 27
**Title and** **abstr-act**	1a	Identification as a randomized trial in the title	0(0)	1(20)	0(0)	1(3.7)
	1b	Structured summary of trial design, methods,results, and conclusions (for specific guidancesee CONSORT for abstracts)	11(52.4)	5(100)	1(100)	17(63.0)

**Table 2:** The average reporting percentage for the introduction section of the CONSORT checklist in the 3 core journals.

Secti-on/To-pic	Item No	Checklist item	JTCM Yes(%) N = 21	CJITWM Yes(%) N = 5	CJCMMYes(%) N = 1	Total Yes(%) N = 27
**Introd-uction**						
**Back-groun-d and objec-tives**	2a	Scientific background and explanation of rationale	17(81.0)	5(100)	1(100)	23(85.2)
	2b	Specific objectives or hypotheses	19(90.5)	5(100)	1(100)	25(92.6)

**Table 3:** The average reporting percentage for the methods section of the CONSORT checklist in the 3 core journals.

Section/Topic	Item No	Checklist item	JTCM Yes(%) N = 21	CJITWMYes(%) N = 5	CJCMMYes(%) N = 1	Total Yes(%) N = 27
**Methods**						
**Trial design**	3a	Description of trial design (such as parallel, factorial)including allocation ratio	21(100)	5(100)	1(100)	27(100)
	3b	Important changes to methods after trialcommencement (such as eligibility criteria),with reasons)	0(0)	0(0)	0(0)	0(0)
**Participants**	4a	Eligibility criteria for participants	18(85.7)	5(100)	1(100)	24(88.9)
	4b	Settings and locations where the data were collected	18(85.7)	5(100)	1(100)	24(88.9)
**Interve-ntions**	5	The interventions for each group with sufficientdetails to allow replication, including how andwhen they were actually administered	21(100)	5(100)	1(100)	27(100)
**Outcomes**	6a	Completely defined pre-specified primary andsecondary outcome measures, including howand when they were assessed	21(100)	5(100)	1(100)	27(100)
	6b	Any changes to trial outcomes after the trialcommenced, with reasons	0(0)	0(0)	0(0)	0(0)
**Sample size**	7a	How sample size was determined	0(0)	0(0)	0(0)	0(0)
	7b	When applicable, explanation of any interimanalyses and stopping guidelines	0(0)	0(0)	0(0)	0(0)
**Randomisation Sequence** **generation**	8a	Method used to generate the randomallocation sequence	10(47.6)	5(100)	0(0)	15(55.6)
	8b	Type of randomisation; details of any restriction(such as blocking and block size)	0(0)	1(20)	0(0)	1(3.7)
**Allocation** **concealment** **mechanism**	9	Mechanism used to implement the random allocationsequence (such as sequentially numbered containers),describing any steps taken to conceal the sequenceuntil interventions were assigned	0(0)	0(0)	0(0)	0(0)
**Implementation**	10	Who generated the random allocation sequence,who enrolled participants, and who assignedparticipants to interventions	0(0)	0(0)	0(0)	0(0)
**Blinding**	11a	If done, who was blinded after assignment tointerventions (for example, participants, careproviders, those assessing outcomes) and how	1 (4.8)	1(20)	0(0)	2(7.4)
	11b	If relevant, description of the similarityof interventions	1 (4.8)	0(0)	0(0)	1(3.7)
**Statistical** **methods**	12a	Statistical methods used to compare groupsfor primary and secondary outcomes	12(57.1)	5(100)	1(100)	18(66.7)
	12b	Methods for additional analyses, such as subgroupanalyses and adjusted analyses	0(0)	1(20)	0(0)	1(3.7)

**Table 4:** The average reporting percentage for the results section of the CONSORT checklist in the 3 core journals.

Section/Topic	Item No	Checklist item	JTCMYes(%)N = 21	CJITWMYes(%)N = 5	CJCMMYes(%)N = 1	TotalYes(%)N = 27
**Results**						
**Participant flow**	13a	For each, the numbers of participants who wererandomly assigned, received intended treatment,and were analysed for the primary outcome group	21(100)	5(100)	1(100)	27(100)
	13b	For each group, losses and exclusions afterrandomisation, together with reasons	2(9.5)	4(80)	0(0)	6(22.2)
**Recruitment**	14a	Dates defining the periods of recruitmentand follow-up	18(85.7)	5(100)	1(100)	24(88.9)
	14b	Why the trial ended or was stopped	0(0)	0(0)	0(0)	0(0)
**Baseline data**	15	A table showing baseline demographic andclinical characteristics for each group	21(100)	5(100)	1(100)	27(100)
**Numbers analysed**	16	For each group, number of participants(denominator) included in each analysis andwhether the analysis was by originalassigned groups	21(100)	5(100)	1(100)	27(100)
**Outcomes and estimation**	17a	For each primary and secondary outcome, resultsfor each group, and the estimated effect size andits precision (such as 95% confidence interval)	21(100)	5(100)	1(100)	27(100)
	17b	For binary outcomes, presentation of both absoluteand relative effect sizes is recommended)	0(0)	0(0)	0(0)	0(0)
**Ancillary analyses**	18	Results of any other analyses performed, includingsubgroup analyses and adjusted analyses,distinguishing pre-specified from exploratory	0(0)	1(20)	0(0)	1(3.7)
**Harms**	19	All important harms or unintended effects in eachgroup (for specific guidance see CONSORT for harms	4(19.0)	3(60)	1(100)	8(29.6)

**Table 5:** The average reporting percentage for the discussion section of the CONSORT checklist in the 3 core journals.

Section/Topic	Item No	Checklist item	JTCM Yes(%) N = 21	CJITWM Yes(%)N = 5	CJCMM Yes(%) N = 1	Total Yes(%) N = 27
**Discussion**						
**Limitations**	20	Trial limitations, addressing sources of potentialbias, imprecision, and, if relevant, multiplicityof analyses	1(4.8)	3(60)	0(0)	4(14.8)
**Generalisability**	21	Generalisability (external validity, applicability)of the trial findings	21(100)	5(100)	1(100)	27(100)
**Interpretation**	22	Interpretation consistent with results, balancingbenefits and harms, and considering otherrelevant evidence	21(100)	5(100)	1(100)	27(100)

**Table 6:** The average reporting percentage for the other information section of the CONSORT checklist in the 3 core journals.

Section/Topic	Item No	Checklist item	JTCM Yes(%) N = 21	CJITWM Yes(%) N = 5	CJCMM Yes(%) N = 1	Total Yes(%) N = 27
**Other information**						
**Registration**	23	Registration number and name of trial registry	0(0)	1(20)	0(0)	1(3.7)
**Protocol**	24	Where the full trial protocol can be accessed,if available	0(0)	0(0)	0(0)	0(0)
**Funding**	25	Sources of funding and other support(such as supply of drugs), role of funders	7(33.3)	4(80)	0(0)	11(40.7)

### Assessment of Reporting Quality

Assessment of all included trials was carried out, and the results were entered directly into a preformatted Excel spreadsheet. Each item was assigned a yes (Y, scored as 1) or no (N, scored as 0) response depending on whether it was reported by the author and each item was weighted with equal importance. A total quality of reporting score, the CONSORT score, was calculated by simply summing the scores of the 37-item checklist, resulting in a possible range of 0–37. Thus, the maximum possible score was 37 points.

Each of the study articles was then independently scored by two investigators. A final score for each item on the checklist was recorded for each article after consensus was reached through discussion between the two or in some cases, after arbitration by a third investigator.

The articles were grouped by journal. The primary outcome of the study was the percentage of applicable items on the CONSORT checklist that were reported in each journal. The main secondary outcome was the percentage of articles that reported each applicable section on the checklist. For clarity, the table has been divided into six sections (title and abstract, introduction, methods, results, discussion and other information) representing where the respective items would be expected to be reported. We compared the total mean number and the percentages reported regarding the breakdown of scores for each section of the CONSORT checklist between the three journals.

PRISMA checklist is provided in Appendix 1(Checklist S1).

### Statistical Analyses

We conducted a descriptive statistical analysis of all evaluated articles. Data were analyzed using Microsoft Excel 2003. Interrater reliability was not calculated by consensus, and agreement on interpretation of items was carried out in a continual manner, such that any reliability analyses would be meaningless and confounded by learning effects between assessors. All 27 relevant studies were checked for compliance with the statement by assessing the fulfillment of the 37 CONSORT items. In order to assess adherence to the CONSORT checklist items, we calculated the number and proportion of reports describing each of the 37 items. In addition, we calculated the number and proportion of these items by the RCTs published in a journal. The sum of the scores was converted to a percentage value for each trial, each journal, each item, each section, and the total of the CONSORT checklist.

For each article, the quality of its reporting was determined by the total number of items it included in the 37-item checklist. For example, a RCT reporting 20 of the 37 items on the checklist would score 54.1%. Each item on the checklist was also evaluated by tabulating the number of RCTs that reported the item. For example, if 23 of 27 RCTs reported item 2a on the checklist, that item would score an overall compliance score of 85.2%.

## Results

**Figure 1:**
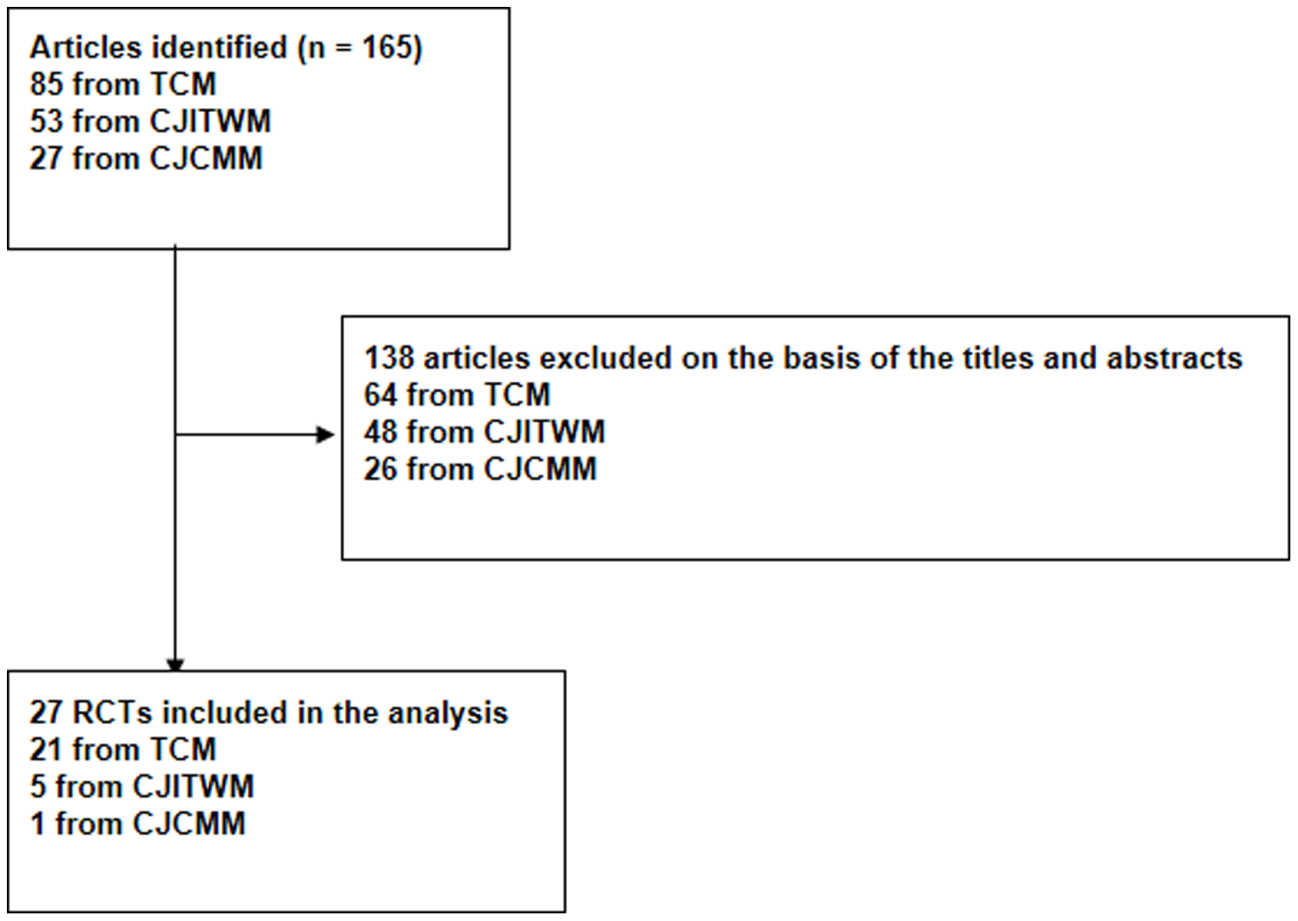
Study selection. Figure 1 shows the flow diagram of the selection of RCTs on the treatment of diabetes mellitus with TCM published in 2011 of the three core journals.

A flow chart of the selected RCTs is shown in Figure 1. The search strategy was chosen to be highly sensitive and less specific, allowing a wide range of initial references which were later reduced by reviewing the titles and abstracts. The CNKI database search identified 165 RCTs published between Jan. 1, 2011 and Dec. 31, 2011 in 3 journals. After reviewing these articles, 27 trials fulfilled the eligibility criteria and were assessed for this study. For this analysis, the Journal of Traditional Chinese Medicine contributed the majority of articles which met the eligibility criteria (21)[9]–[29], followed by the Chinese Journal of Integrated Traditional & Western Medicine (5)[30]–[34], and the China Journal of Chinese Materia Medica (1) [35].

Table 7 shows the sources of the articles.

**Table 7:** Sources of the articles (% of articles, N = 27).

	JTCM	CJITWM	CJCMM
**Articles (%)**	21(77.8%)	5(18.5%)	1(3.7%)

There were no disagreements between investigators regarding identification of articles that did not meet these criteria.

Table 1, Table 2, Table 3, Table 4, Table5 and Table 6 show the total mean number and the percentages of the trials that reported the CONSORT items in the 6 different sections, respectively. Table 1, Table 2, Table 3, Table 4, Table 5 and Table 6 also show the mean number and percentages of CONSORT items reported by journal category and compare the extent to which RCTs published in journals reported each of the 37 items examined. The highest possible number for each item was the number of trials included in each journal or the total number of trials.

Table 8 shows the average reporting percentage for each section and the total of the CONSORT checklist in the 3 core journals. The report quality assessed using the CONSORT criteria varied from 0 to 37 (maximum score was 37).

**Table 8:** The average reporting percentage for each section and the total of the CONSORT checklist in the 3 core journals.

	JTCM	CJITWM	CJCMM	Total
**Section**	**Yes(%)**	**Yes(%)**	**Yes(%)**	**Yes(%)**
	**N = 21**	**N = 5**	**N = 1**	**N = 27**
**title and** **abstract**	26.2	60.0	50.0	33.3
**introduction**	85.7	100.0	100.0	88.9
**methods**	34.5	44.7	35.3	36.4
**results**	51.4	66.0	60.0	54.4
**discussion**	68.3	86.7	66.7	71.6
**other** **information**	11.1	33.3	0.0	14.8
**total percentage(%)** **of N = 37**	42.2	56.8	46.0	45.0

* JTCM: the Journal of Traditional Chinese Medicine,

CJITWM: the Chinese Journal of Integrated Traditional & Western Medicine,

CJCMM: the China Journal of Chinese Materia Medica,

Yes (%): Number (%) of trials in which the item was reported.

As seen in Table 1, Table 2, Table 3, Table 4, Table 5 and Table 6, only ten items were reported by 90% or more of the included trials. These included specific objectives or hypotheses, description of the trial design, the interventions for each group, defined outcome measures, the number of participants in each group, baseline data, numbers analyzed, generalizability (external validity, applicability) of the trial findings, outcomes and estimation, and the interpretation.

In contrast, none of the trials reported important changes to methods after trial commencement (such as eligibility criteria), any changes to trial outcomes after the trial commenced, how sample size was determined, explanation of any interim analyses and stopping guidelines when applicable, who generated the random allocation sequence, who enrolled participants, and who assigned participants to interventions, allocation concealment mechanism, why the trial ended or was stopped, where the full trial protocol can be accessed (if available), and presentation of both absolute and relative effect sizes which is recommended for binary outcomes. In addition, none of the trials included a flow diagram.

In addition, a number of items identified by data extraction were reported by only a small percentage of the trials. For example, only 1 (3.7%) stated in the title that the trial was randomized, only 1 (3.7%) reported the type of randomization; details of any restrictions (such as blocking and block size), only 2 (7.4%) reported blinding, only 1 (3.7%) described the similarity of interventions (if relevant), only 1 (3.7%) reported the methods for additional analyses (such as subgroup analyses and adjusted analyses), only 6 (22.2%) reported the losses and exclusions after randomization for each group (together with reasons), only 1 (3.7%) reported the ancillary analyses, only 8 (29.6%) reported the harms, only 4 (14.8%) reported the trial limitations, only 1 (3.7%) reported the registration number and name of the trial registry, and only 11 (40.7%) reported the sources of funding and other support.

Table 8 summarizes the average reporting percentages of the breakdown of scores for each section of the CONSORT checklist for the included trials. As seen in Table 8, the average reporting percentage for the “title and abstract” section of the trials for the 3 core journals (the Journal of Traditional Chinese Medicine, the Chinese Journal of Integrated Traditional & Western Medicine, and the China Journal of Chinese Materia Medica) was 26.2%, 60.0%, and 50.0%, respectively, these values for the “introduction” section were 85.7%, 100.0%, and 100.0%, respectively, for the “methods” section they were 34.5%, 44.7%, and 35.3%, respectively, for the “results” section they were 51.4%, 66.0%, and 60.0%, respectively, for the “discussion” section they were 68.3%, 86.7%, and 66.7%, respectively, and for the “other information” section they were 11.1%, 33.3%, and 0.0%, respectively. The introduction, results and discussion sections showed the highest scores with respect to their maximum theoretical scores.

## Discussion

Chinese herbal medicine is becoming increasingly popular in industrialized nations as a form of “alternative” or “complementary” medicine. In order that this type of medicine is fully integrated into conventional medical systems, thereby utilizing its considerable benefits, evidence of the safety and efficacy of herbs and herbal products is necessary. Both randomized controlled trials (RCTs) and systematic reviews are commonly thought to provide the strongest level of evidence regarding the treatment efficacy of competing therapeutic interventions. The credibility of the evidence to support a treatment approach such as Chinese herbal medicine therefore depends on the quality of RCTs [36].

China is a developing country and has many patients with diabetes mellitus, therefore it is necessary to carry out more research in this field. However, fewer studies in this field have been published in foreign journals, as funding sources and the opportunity of collaborative research with foreign researchers are still limited. While English-speaking countries have led this movement to include standardization, there is still a limited amount of information from non-English-speaking countries, including China.

Although surveys and studies similar to the present study have previously been conducted, to our knowledge, this research is the first to investigate the quality of reporting of RCTs with particular reference to CONSORT for the treatment of diabetes mellitus with TCM published in the Chinese language following the revised CONSORT Statement (2010 version). Zhao-Xiang BIAN et al [36] carried out a systematic review on type 2 diabetes mellitus reported in 66 RCTs, the largest number of RCTs on Chinese herbal medicine (CHM) in the Cochrane Library (up to July 2005), and recorded the quality of reporting in RCTs of CHM. All studies were published in English. This study which assessed the quality of the reporting of clinical trials in type 2 diabetes mellitus concluded that “the overall quality of reporting of RCTs of CHM evaluated with a revised CONSORT (2001 version) checklist was poor” [36].

Therefore, in the present study, we investigated the quality of reporting of RCTs published in three leading Chinese medical journals regarding the treatment of diabetes mellitus with TCM using the recent CONSORT (2010 version) statement. The relevant content of each reviewed study was matched to the CONSORT statement to assess its compliance, to determine which sections of the articles should be improved, and we attempted to provide guidelines on how to make these improvements.

There are many other tools available to evaluate the quality of RCTs. We chose the CONSORT checklist for several reasons. Firstly, CONSORT is officially supported by the World Association of Medical Authors and the International Committee of Medical Journal Editors. Secondly, several reports demonstrated that the quality of reporting could be improved if the CONSORT statement was followed more closely[37]–[40]. Thirdly, CONSORT has been applied in many other medical specialities[41]–[43]. We used the CONSORT checklist 2010 version as it allowed us to compare our results with those obtained in other medical specialities and therapeutic areas.

The CSCD (Chinese Science Citation Database) contains the most authoritative citation reports in China, it is updated every two years and has achieved a good reputation in China. The CSCD is divided into the core journals and the expansion of journals. Chinese authors consistently select these journals for publication of their research papers as well as degree papers.

In the present study, 3 core journals were selected from CSCD-indexed journals to investigate adherence to the CONSORT statement by these high-impact Chinese medical journals. To determine whether a journal was a CONSORT endorser, we searched the “instructions to authors” section on the journal’s website to assess whether the standard CONSORT guidelines were endorsed as part of the publication process. We found that none of the 3 journals mentioned the CONSORT statement in their “instructions to authors” even though the CONSORT Statement was first introduced into China in 1997. It was not stated in the instructions for authors that clinical trials should have trial registration numbers and that priority would be given to clinical trials which were registered.

The results of our study show that the reporting quality of RCTs on the treatment of diabetes mellitus with TCM published in these three journals was insufficient to allow readers to assess the validity of the trials. Overall compliance with CONSORT guidelines in our study was 45.0%, and very few papers complied with all the criteria. These results come as no surprise when looking at similar studies published in the medical literature[36]–[45]. He et al concluded that the quality of the current TCM RCTs as judged by their publications is generally poor, especially those published in Chinese journals [38].

In addition, Hopewell S et al. confirmed that the reports on many randomized trials, most of which are published in specialty and subspecialty journals, have crucial information missing or reported in such a way that the information cannot be understood and/or used by readers [39]. Francisco Dasí et al concluded that “even high-impact journals publish articles of unsuitable quality” [40].

David Moher et al concluded that “While improvements have been seen over time, the majority of trials still do not report essential information available from every trial such as full details of the interventions and outcomes, and the methods of allocation of interventions” [46].

Although the articles were published in high-impact index journals, the results of this study show that the title and abstract, methods, and other information sections showed lower scores with respect to their maximum theoretical scores, which indicated that some aspects of the articles require improvement. We found that improvements are particularly needed in the “methods” section. Authors should pay particular attention to providing a detailed description of randomization, blinding, allocation concealment, and conducting proper statistical tests, including intention-to-treat analysis where appropriate. The discussion section in most articles was strong. In particular, authors provided a good literature review and a perspective on how their study impacts on the area of research. However, few authors attempted to generalize their findings in order to help clinicians determine whether the findings applied to their patients. This issue is particularly important because it has been reported that studies with lower methodological quality are most likely to produce “positive results” and are therefore more likely to be published [47].

A striking observation was that the trials were infrequently identified as randomized in the title. The ability to identify a report of a randomized trial in an electronic database depends to a large extent on how it was indexed. Indexers may not classify a report as a randomized trial if the authors do not explicitly report this information. To help ensure that a study is appropriately indexed and easily identified, authors should use the word “randomized” in the title to indicate that the participants were randomly assigned to their comparison groups [8].

To improve the reporting quality of abstracts of RCTs, the CONSORT group previously suggested a 16-item list which should be addressed in the abstract of a given published RCT [8]. Good reporting in RCT abstracts is crucial because they may have a significant and immediate impact on patient care. They must provide appropriate information to allow readers to quickly assess the validity and applicability of the findings regarding the care of their individual patients and to decide whether they should seek more information regarding a trial. However, our analysis revealed that issues such as randomization, blinding, harms, trial registration and funding were still underreported in the abstracts of RCTs published in these three journals. However, limited available space should be considered in this regard. Depending on journal requirements, the word count is usually limited to 250 to 300 words for abstracts. Therefore, the abstract format is often strictly controlled in terms of objective, methods, results, and conclusions. Thus, investigators are influenced and guided by these requirements.

Approval of the RCT by a clinical research ethics committee (CREC) and obtaining informed consent from patients should be mandatory conditions in carrying out a RCT and could, therefore, be considered the main components of ethical research in humans. Therefore, both ethical and methodological aspects should be listed in detail in articles, which would help the reader to make a proper assessment of a RCT [48]. In our study, the reporting of ethical issues was inadequate. None of the RCTs reported having ethical committee approval, although the latter is a legal requirement in China. In addition, a few studies mentioned that the participants attended of “their own free will” but the remainder made no mention of consent.

Similarly, trial registration is stipulated by the CONSORT guidelines and the World Health Organization states that the registration of all interventional trials is a scientific, ethical and moral responsibility. In 2004, the International Committee of Medical Journal Editors (ICMJE) implemented a policy change to state that they would only consider trials for publication if they had been registered before the enrolment of the first participant. This resulted in a dramatic increase in the number of trials being registered, and an associated improvement in the quality of reporting for registered trials [49]. However, only 1 (3.7%) RCT in our sample reported a trial registered with a recognized database and stated the registration number. This may have been due to lack of compulsory policies that only registered trials can be published in journals in China. Now, the Chinese Periodicals Association has only just recommended that clinical trials which have been registered should be prioritized for publication.

Many studies have shown that RCTs not using randomization, allocation concealment or blinding exaggerate estimates of effect to varying degrees. Compared with RCTs using blinding, RCTs without blinding yield larger (17%) estimates of treatment effects and in trials with subjective outcomes, effect estimates are exaggerated by 25%. Compared with the RCTs using adequate allocation concealment, RCTs using unclear or inadequate concealment of allocation exaggerate estimates of effect by 30%–41% [50]–[54].

Our study also showed some disappointing results. For example, with regard to randomization, none of the trials reported detailed information on their allocation concealment mechanism and implementation. The process of randomization was poorly explained in most studies. None of the studies stated who generated the allocation sequence and whether the same person enrolled the participants. In our opinion (and that of others), a marked improvement in the items relating to randomization was found [55]. Just over half the studies (n = 15) fully described the methods used to generate the allocation sequence. It is well known that only an unpredictable and unknown allocation schedule could minimize selection and confounding biases. Therefore, the CONSORT Statement deemed that use of the term ‘randomized’ is not sufficient. These reports showed that compared with other “flaws”, unclear or inadequate allocation concealment will cause a larger bias, which highlights the importance of allocation concealment. He et al [38] reported that adequate allocation concealment was the smallest proportion (7%) of the aspects they assessed, and some investigations have shown that only 6.8% of the RCTs published in Chinese journals were deemed authentic randomized trials [56]. Thus, the quality of RCTs on TCM in this study may be overstated.

Blinding is an important safeguard against bias, particularly when assessing subjective outcomes [8]. Regardless of whether blinding is possible, authors can and should always state who was blinded (that is, participants, healthcare providers, data collectors, and outcome adjudicators). Unfortunately, in total, 25 papers (92.6%) provided no information on blinding of either participants or investigators. We acknowledge that blinding is particularly challenging in the treatment of diabetes mellitus with TCM, as blinding of participants and investigators is often difficult or impossible, however, blinding of data collectors and outcome adjudicators is often achievable. If blinding is not achieved, this should be mentioned and discussed as a potential bias.

Furthermore, although all articles reported on the number of patients, details on the method used to calculate the sample size (power calculation) were not described in any of the articles. Reporting of a priori sample size calculation is an indicator of adequate trial planning and indicates whether a trial ends earlier than planned. However, Herdan A. et al [55] reported that sample size calculation showed a sharp increase over time, i.e. the percentage of trials correctly reporting this item increased from 23% (three of 13 reports) to 71% (17 of 27 reports) in the post-CONSORT period. Another concern in all these studies was the lack of reported information on the flow of participants through each stage of the treatment protocol, including information regarding the number of care providers or centers performing the intervention. The original CONSORT statement strongly advocated a flow diagram in order to follow the flow of patients from recruitment to the end of the clinical trial [8].

In our review, only nine papers (33.3%) did not report which statistical methods had been used. A total of 2 papers (7.4%) presented p-values, and 3 papers (11.1%) presented a 95% CI of the treatment effect. The statistical methods used to compare groups were reasonably well described, but in most cases the general statistical approach was described rather than linking a specific test to an outcome. We did not determine whether the statistical test was appropriate, although many were deficient in describing confidence intervals and accounting for multiple testing. In addition, all papers merely reported an ordered list of results from which it was not possible to distinguish which outcome was the primary outcome. In the reviewed trials, most papers only described baseline characteristics of the participants using text in the baseline information, simply stating that the baseline characteristics matched in both arms instead of fully reporteing the baseline characteristics of the participants in a separate table.

Identification and discussion of the weaknesses of a study are particularly important and are needed for a balanced interpretation of the results. Only 14.8% of RCTs studies discussed the limitations of the study. Similarly, information on the harms as well as the benefits of interventions is stipulated in the CONSORT guidelines. However, details of harms and unintended effects were reported in just 29.6% of the RCTs in our sample. Reporting of adverse events should always be considered during the study design as data on adverse events are less susceptible to bias and confounders when they are collected prospectively rather than retrospectively. Editors should probably state that authors should be encouraged to report undesired effects. Proper definition and reporting of adverse events are crucial for critical appraisal of the study results and facilitates systematic reviews and meta-analyses.

A shortcoming in all of the included studies was the lack of dates defining the periods of follow-up. In total, 24 papers (88.9%) reported the date on which participants were recruited, however, there was no information provided on the length of time the participants were followed. The length of follow-up is not always a fixed period after randomization. In many RCTs in which the outcome is time to an event, follow-up of all participants is ended on a specific date. This date should be provided, and it is also useful to report the minimum, maximum and median duration of follow-up. In addition, another important area of RCT quality is related to loss to follow-up. The majority of the trials reported that none of the participants had dropped out. In the reviewed trials, 77.8% of studies failed to report drop-out rates, which revealed that Chinese researchers do not adequately consider this problem. In general, the higher the ratio of participants with missing data to participants with events, then the greater the potential for bias. Perhaps for this reason many studies were unwilling to report real drop-out rates. When studies have drop-out rates greater than 20%, all results are likely to lose their authenticity, and consequently, clinical value will be badly affected if not meaningless.

It is important that authors describe in detail the role of the funding bodies and their level of involvement and influence on design, conduct and analysis of the data. A total lack of information on economic studies was observed in our study. This economic information is particularly important in a medical system in which many clinical decisions rely on tools such as the economic evaluation and the intervention outcome. Of the 27 papers, only 11 (40.7%) reported their sources of funding. Funding was from provincial/municipal and national sources (including provincial/municipal sources) in 8 trials (29.6%) and 3 trials (11.1%), respectively. Although there was some improvement over time, this improvement was only moderate.

In compliance with the legal regulations, the promoter of a RCT is required to publish the results (both positive and negative) in scientific journals, with reference to the clinical research ethics committee that approved the study. However, in our study, none of the trials published negative results. It has long been known that only a small proportion of RCTs publish negative results in scientific journals, which seems to be a common problem as the publication of RCTs in different countries varies between 31% and 67% [57]. In a review article, von Elm et al [58] concluded that the main reason for this was lack of time, however, some researchers responded that negative results were the major cause of nonpublication. The selective publication of “statistically significant” results is well documented, and it has been observed that a study was more likely to be published if the results were positive [59], [60].

Therefore, there is considerable room for improvement in the publication of RCTs conducted in China. In particular, the publication of methods described in as much detail as possible and the titles and abstracts and other information regarding the study should be improved.

### Limitations

Considering the rigor of this systematic evaluation, we are confident that it is a complete summary of all available evidence. However, several limitations of the study should be acknowledged. First, since we only assessed the RCTs published by the three CSCD-indexed, leading Chinese medical journals from January to December 2011, the results do not fully represent the reporting quality of all Chinese RCTs. Second, we only assessed the reporting quality of RCTs, not the trials themselves, and failure to report is not necessarily equivalent to failure to actually carry out the procedures. It is possible that a poorly reported study was well designed and executed, and a well-reported study may have had several shortcomings. However, transparent and clear reporting is likely to be the result of a well-designed trial allowing the reader to make a judgment. Third, we did not calculate the Kappa statistic to quantitatively measure the interobserver agreement. Fourth, it should be noted that some journals have more restrictions than others in terms of word count for example. These restrictions may be related detailed descriptions of eligibility criteria, abstract, process of consent acquisition, and blinding methods. Fifth, the time frame (from January to December 2011) chosen for this review was meant to provide an indication of CONSORT reporting for the selected year only, and may not be representative of the journals’ reporting in general. In addition, any improvements in CONSORT reporting may take some time to become apparent after official endorsement of the CONSORT statement. Hence, for this review, the one year interval may not provide a sufficient time frame to observe dramatic changes in the reporting of RCTs. Sixth, the CONSORT score, although based on the statement, requires to be validated by other investigators. Although the exact score a study received may not be reproduced if evaluated by others, this study indicates the wide variation in compliance with the CONSORT statement. For example, each item was assigned a yes (Y, scored as 1) or no (N, scored as 0) response depending on whether the item was reported by the author. It may be unfair if an item was scored as 1, if it included three conditions (appropriately and transparently presented, inadequately stated, unclear stated); while an item was scored as 0, if it included two conditions (not applicable, not mentioned). The CONSORT statement itself has been revised, thus future compliance of RCTs needs to be amended. Seventh, the journals included in this study were selected due to their high impact factor. However, there are many other journals which should be assessed for their reporting of CONSORT information if a more comprehensive evaluation is undertaken. Eighth, criteria for the CONSORT statement were not weighted for scoring purposes, based on the assumption that each item of the CONSORT statement had equal importance. We avoided weighting as it would be arbitrary and subjective and thus subject to criticism. Another potential limitation is that we assessed only publications in Chinese, which may have introduced publication bias. However, only 10% of the eligible articles were reported in other languages, and it is unlikely that their inclusion would have changed the overall results.

## Conclusions

In summary, the reporting quality of Chinese RCTs on the treatment of diabetes mellitus with TCM published in these three leading medical journals is far from satisfactory, particularly with respect to the title and abstract, methods and other information sections. The methodological quality of RCTs in China did not show any apparent improvement since the Chinese State Food and Drug Administration (SFDA) recommended Good Clinical Practice (GCP) in 1998. More attention should be paid to the design and methodology of these trials. Specifically, items such as identification as a randomized trial in the title, allocation concealment, blinding, sample size calculation, a participant flow diagram, information on the harms or unintended effects, trial limitations, trial registration, ethical committee approval, the patient’s informed consent, the periods of follow-up, role of funders, and compliance of participants and investigators should be implemented in all RCTs to improve their overall quality and ensure the validity and usefulness of their results.

Editorial mechanisms can be very successful in improving the quality and completeness of the research evidence by enforcing the requirements for article submission. The results of this review should strongly encourage journal editors to change the instructions to authors to ensure that the issues that affect the understanding of a manuscript, by referees or readers, and how the study was undertaken are adequately described. There is good evidence in the literature that the adoption of CONSORT improves the quality of both the conduct and reporting of trials in journals that have taken the decision to make it a requirement for submission acceptance[39], [61]–[63]. Therefore, authors, editors and referees should adopt the CONSORT Statement to improve the reporting quality of Chinese RCTs and ensure truth and reliability of conclusions. In the interest of transparency, authors of trial reports should be encouraged to state why any item in the checklist was not reported, thus making the reader’s task of understanding conformity to CONSORT more straightforward.

## Supporting Information

Checklist S1 **PRISMA checklist.** PRISMA 2009 checklist of information to include when reporting a systematic review.
(DOC)
